# Regulation of R-Loops in DNA Tumor Viruses

**DOI:** 10.3390/pathogens13100863

**Published:** 2024-10-02

**Authors:** Anaiya Crowner, Keely Smith, Marsha DeSmet

**Affiliations:** 1 Indiana University Simon Comprehensive Cancer Center American Cancer Society Post-Baccalaureate Diversity in Cancer Research Education Program, Indiana University School of Medicine, Indianapolis, IN 46202, USA; 2 Department of Dermatology, Indiana University School of Medicine, Indianapolis, IN 46202, USA

**Keywords:** DNA tumor viruses, R-loops, SETX, replication, transcription, oncogenesis

## Abstract

R-loops are triple-stranded nucleic acid structures that occur when newly synthesized single-stranded RNA anneals to duplex DNA upon the collision of replication forks with transcription complexes. These RNA–DNA hybrids facilitate several transcriptional processes in the cell and have been described extensively in the literature. Recently, evidence has emerged that R-loops are key regulators of DNA tumor virus transcription and the replication of their lifecycle. Studies have demonstrated that R-loops on the Human Papillomavirus (HPV) genome must be resolved to maintain genome maintenance and avoid viral integration, a hallmark of HPV cancers. For Epstein–Barr virus (EBV), R-loops are formed at the *oriLyt* to establish lytic replication. Structural maintenance of chromosome proteins 5/6 (SMC5/6) bind to these viral R-loops to repress EBV lytic replication. Most viruses in the *herpesvirales* order, such as KSHV, contain R-loop-forming sequences. In this perspective, we will describe the current, although limited, literature demonstrating the importance of RNA–DNA hybrids to regulate DNA virus transcription. We will also detail potential new areas of R-loop research and how these viruses can be used as tools to study the growing field of R-loops.

## 1. Introduction

R-loops are triple-stranded nucleic acid structures that form during transcription. An R-loop is composed of a shifted single-stranded DNA and a newly synthesized single-stranded RNA that anneals to template DNA [1,2]. RNA–DNA hybrids form transiently near transcription initiation and termination sites to facilitate RNA polymerase II pausing [3]. R-loops on the human genome range from 200 to 500 bp in length [4]. R-loops can occur in an “unscheduled” manner, leading to potential DNA damage, genomic instability, and transcription elongation effects. In contrast, there are regulatory, “scheduled” R-loops that are formed to terminate transcription and promote DNA repair [2]. Replication and transcription complex collisions can occur on the genome, creating R-loops capable of impeding replication. It is estimated that 260 R-loops occur in a human cell per day; failure to resolve R-loops may lead to a DNA damage event [5]. To prevent accumulation, helicases and RNases are recruited to these complexes for resolution [6]. Human Senataxin (SETX) and its yeast homolog Sen1 were the first described RNA–DNA helicases involved in R-loop resolution [7,8]. Senataxin and Sen1 associate with replication forks and facilitate their movement through RNA polymerase II (RNAPII) transcription bubbles [9]. SETX also interacts directly with RNAPII and has been implicated in DNA strand resolution during fork repair [10,11]. Complementing this process, ribonuclease H (RNase H) executes the targeted cleavage of RNA within R-loops for efficient resolution [12]. Eukaryotes have two classes of RNase H enzymes, each capable of RNA elimination within the R-loop: RNAse H1 and RNase 2. The enzyme RNAse H2 is necessary for R-loop removal during G2/M and ribonucleotide excision repair during S-phases of the cell cycle. In contrast, RNAase H1 activity is not restricted to cell cycle [13]. Under experimental conditions, RNAse H1 overexpression is often used to eliminate cellular R-loops [14]. R-loops control various biological processes in the mammalian cell. In this review, we will describe an emerging new function of R-loops: as key regulators of DNA tumor virus replication, transcription, and carcinogenesis. Researchers first described viral R-loops over 30 years ago, using them as tools to map viral transcripts. Recent studies, although limited, have begun to identify the importance of R-loops in DNA virus episomal maintenance and as DNA damage initiators to drive carcinogenesis. Experimental methods have evolved to allow the more precise identification of R-loop sites within the genome. DNA–RNA immunoprecipitation (DRIP) followed by sequencing or qPCR using primers for specific regions in the viral genome is a commonly used method for R-loop mapping [15,16,17]. In this perspective, we will highlight recent RNA–DNA hybrid discoveries made with DNA tumor viruses and discuss the future of this emerging field.

## 2. Human Papillomavirus (HPV)

HPV is a double-stranded DNA tumor virus associated with various cancers. Among the most prevalent are those affecting epithelial and mucosal tissues, such as cervical, oropharyngeal, and anal cancers. Structurally, HPV is composed of distinct early and late regions, with the early regions being implicated in its oncogenic potential. There are low-risk (LR) and high-risk (HR) HPVs [18]. Low-risk HPVs can result in common and flat warts while high-risk HPVs are associated with cancer progression, including squamous cell carcinoma (SCC) and adenocarcinoma (AC) [18]. The early genes E6 and E7 play a vital role in the oncogenic transformation processes associated with HR HPVs [19]. The HR E6 protein forms a complex with the tumor suppressor p53 for the rapid proteasome-mediated degradation of p53 [20]. HR E7 proteins interact with and promote the degradation of the retinoblastoma tumor suppressor protein, pRB [21]. These oncogenic viral protein–host interactions disrupt key regulatory pathways within host cells, promoting the uncontrolled proliferation and evasion of cellular defense mechanisms. Furthermore, recent research focusing on the pathogenicity of HPV suggests an interplay between the HPV oncogenes and R-loop formation [22,23].

Enriched mammalian genome R-loops, between 50 and 500-fold higher, were detected in HPV-31-positive keratinocytes, CIN612 cells, on ALU repeat elements and regulatory elements [22,24]. Although there was an increase in global R-loops, there was no effect on global transcription in the HPV-positive cells compared to normal keratinocytes [24]. Most of the genes enriched for R-loops in CIN612 cells were involved in cancer and DNA virus infection. In normal keratinocytes, the genes involved in clathrin binding, kinase activity, and lipid binding were identified as R-loop-containing genes [24]. RNA sequencing (RNA-seq) analysis discovered that RNase H1 overexpression enhanced the expression of genes involved in innate immunity [22]. HPV-E6, not HPV-E7, induced R-loop formation on the human genome in keratinocytes. This mechanism is likely through the E6-mediated depletion of p53 since a reduction in p53 in E7-expressing keratinocytes also enriched R-loops [22]. This is not surprising, as p53 was recently identified as a key regulator of R-loops and genome integrity. In p53-deficient cells, the levels of S-adenosylmethionine (SAM) were deficient and caused heterochromatin depression and the upregulation of satellite RNAs, leading to R-loops [25]. These studies show a novel mechanism in which E6 induces host R-loops and genomic instability.

R-loops were characterized on the HPV genome as early as 1987, when Chow et al. identified and mapped the R-loops of RNAs isolated from plantar warts [26]. R-loops were also detected on the HPV p97 and early poly A gene regions [22,23]. The R-loop-resolving factors BRD4 [27], RNase H1 [22], SETX [23], and TopoI [28,29] have all been detected on the HPV genome. The depletion of the R-loop-resolving factor RNase H1 increased viral R-loops and reduced HPV episomes and the copy number in HPV-31 episomal CIN612 cells [22]. The depletion of RNase H1 suppressed E1, E6, and E7 transcripts. Interestingly, the over-expression of RNase H1 also reduced HPV genome maintenance and viral transcripts in CIN612 cells [22].

SETX depletion increased R-loop detection on the viral p97 promoter and decreased the viral transcripts in episomal CIN612 and HPV-16 episomal W12 cells [23]. SiRNAs to Aquarius (AQR), an RNA helicase found to resolve mammalian R-loops [30], had no effect on the R-loop quantities in these HPV episomal cell lines [23]. These results strongly support that SETX is a helicase responsible for R-loop resolution on the HPV genome.

The PV activator E2 and E1 DNA helicase proteins are the first proteins to be expressed by all HPVs [31]. Basal levels of E2 are required for the initiation of HPV replication [32,33]. The E2 protein is composed of an approximately 220 amino acid transactivation domain (TAD), a hinge region, and a DNA-binding domain (DBD), which recognizes the inverted palindrome ACCN6GGT sequence in the PV genome, including its origin of replication (ori) [34]. The E2 protein is necessary for the replication of the HPV genome, the activation of the viral early promoter for transcription, and the tethering of viral genomes to host mitotic chromosomes in basal cells during cellular division. HPV E2 was found in complex with SETX. This experiment was performed in the presence of the nuclease Benzonase to eliminate the possibility of each protein independently binding to the same DNA region and co-immunoprecipitating together [23]. Using the HPV-31 p97-promoter upstream of the firefly luciferase construct, SETX overexpression alone had a modest effect on promoter activity. However, the overexpression of E2 with SETX robustly activated E2-dependent promoter activity [23]. HPV E2 may be necessary for SETX activity on the p97 promoter. It is unclear if E2 helps to recruit SETX to the p97 promoter or if SETX-binding factors like Zpr1 [35,36,37] and BRCA1 [38,39] recruit the helicase to R-loops on the HPV genome.

RNA–DNA hybrids also form during homologous recombination. HPV differentiation-associated amplification has been proposed to utilize homologous recombination to generate unit size circular genomes [40,41]. Templeton et al. [22] observed a significant decrease in R-loops in differentiated HPV-positive keratinocytes, which correlated with increases in the SETX levels but not those of the R-loop-resolving factors RNase H1, Mre11, DDX11 and Topo1. Are SETX levels high to prevent R-loops forming during differentiation-dependent HPV genome amplification? The role of R-loops during this stage of the HPV lifecycle is still unknown.

**Figure 1:**
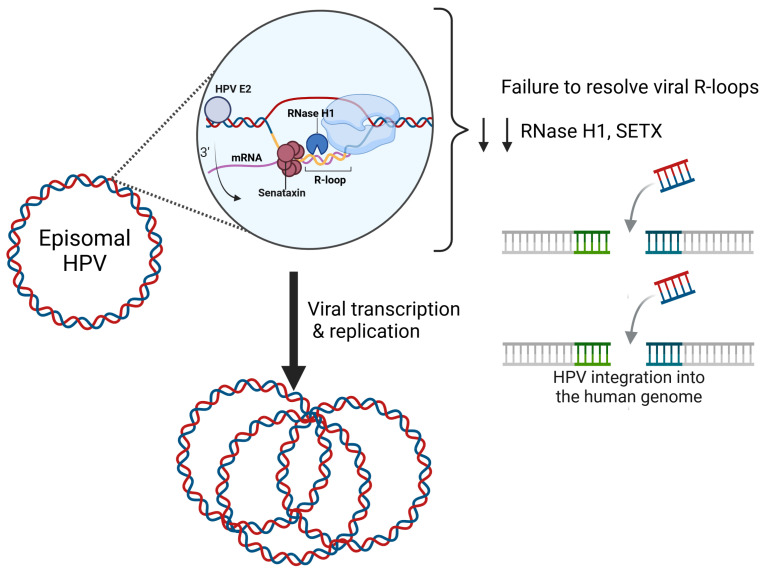
R-loop resolution on the HPV genome is mediated by Senataxin and RNase H1. HPV is a double-stranded 8 kilobase pair DNA genome that replicates as an episome. HPV transcription and replication is mediated through the viral E2 protein. The HPV-E2 protein binds to E2-binding sites throughout the long control region. E2 mediates HPV transcription and replication through host protein–protein interactions. RNA–DNA hybrids form at the p97 promoter on the HPV genome during transcription. RNase H1 and SETX are recruited to the viral genome to resolve these viral R-loops. The depletion of RNase H1 and SETX increases R-loop accumulation, prevents genome maintenance, and increases viral integration. Figure was created with BioRender.com.

What happens if R-loops fail to be resolved on the HPV genome? Failure to remove R-loops may result in the dysregulation of transcription and genomic instability, leading to PV integration, a step towards malignant progression. This hypothesis is supported by several studies. For example, the depletion of RNase H1 increased global R-loop levels and impaired episomal HPV maintenance [22]. Short-term SETX knockdown decreased episomal HPV-31 DNA in CIN612 cells. A subclone population of CIN612 that contains only integrated HPV-31 DNA had significantly fewer R-loops on the p97 promoter compared to CIN612 cells with episomal HPV-31 DNA [23]. These results suggest that R-loop resolution is essential to the HPV lifecycle and necessary for episomal maintenance (Figure 1).

**Figure 2:**
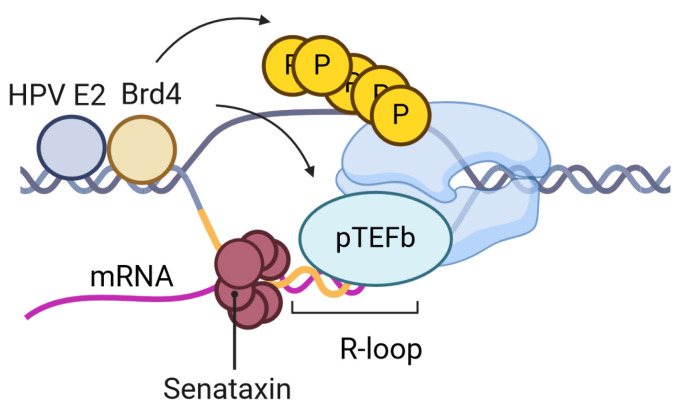
BRD4 activates HPV replication through many mechanisms. BRD4 phosphorylates RNAPII [54] and recruits pTEFb [52,55,56] to transcriptional start sites and to the HPV promoter. Recent studies have identified BRD4 as a key regulator for R-loop resolution on the mammalian genome. Future studies are needed to determine if BRD4 mediates HPV R-loop resolution through pTEFb to prevent RNAPII stalling. Figure was created with BioRender.com.

BRD4 functions as a transcriptional co-activator and is involved in epigenetic regulation. It is well documented that BRD4 is necessary for HPV transcription and replication through its interaction with HPV E2 (reviewed [42,43]). One function of BRD4 and its binding partner pTEFb (composed for Cyclin T and Cdk9) is to stimulate the processivity of RNA polymerase through the BRD4 C-terminus motif (CTM). The depletion of BRD4, using a BRD4 PROTAC, in episomal HPV-31 cells (CIN612) suppressed viral transcripts [44]. P-TEFb was recruited to the HPV episome [45] and pTEFb inhibition reduced but did not block E2-dependent transcription [46]. These results are consistent with BRD4-mediated HPV transcriptional processivity [46]. Brd4 mediates R-loop resolution [47,48,49]. The loss of BRD4 results in the accumulation of mammalian R-loops [50]. The BRD4 CTM is necessary to prevent R-loop DNA damage [51] and to stimulate Pol II pause release [52]. This domain is the same domain of BRD4 that binds CDK9 to phosphorylate Serine 2 on the tail of RNAPII. The treatment of HEK293T cells with 5,6-dichloro-1-β-D-ribofuranosylbenzimidazole (DRB), a CDK9 inhibitor, increases R-loop formation [53]. BRD4 is essential for mammalian R-loop resolution and is likely to mediate this process on the HPV genome (Figure 2).

## 3. Epstein–Barr Virus (EBV)

R-loops were first detected for Epstein–Barr virus (EBV) at the EBV origin of lytic replication (OriLyt) [57]. EBV is a herpesvirus associated with human cancers such as Hodgkins’s lymphoma, Burkitt’s lymphoma and nasopharyngeal carcinomas [58]. The EBV genome is maintained extrachromosomally in infected cells [59]. During latent infection, EBV replication is mediated through the *OriP* composed of the dyad symmetry (DS) and family of repeats (FR) elements during the S phase. Replication during EBV’s lytic cycle is mediated through another origin, the *oriLyt*. Lytic replication is dependent on the Zta and BALF2 transcription factors [59]. The *oriLyt* is approximately 7700 base pairs and contains two genes, BHLF1 and BHRF1. BHLF1 codes for the viral basic leucine zipper protein Zta and is important for latent viral replication and B cell immortalization [60].

Using a series of response element deletions in the EBV genome, Rennekamp et al. [57] identified that BHLF1 RNA transcription is essential for replication from the *oriLyt.* RNA–DNA hybrids were detected at the *oriLyt* in the early stages of lytic replication using a specific RNA–DNA hybrid antibody, and these R-loops were eliminated over time or with RNase H1 treatment [57]. RNase H1 treatment impaired latent EBV replication but had no effect on HSV1 replication [57]. Rennekamp et al. [57] went on to demonstrate that BALF2 but not Zta *oriLyt* association was impaired in the presence of RNase H1. These data suggest that the BHLF1 RNA forms R-loops at the *oriLyt* to mediate BALF2 binding to the latent origin for replication.

Structural maintenance of chromosome (SMC) complexes bind to DNA for the condensing and protection of chromosomes [61]. The SMC5/6 cohesion complex forms a ring structure to entrap DNA. SMC6 was identified as a protein that interacts with RNA/DNA hybrids [62]. In yeast, SMC5/6 prevented the accumulation of toxic homologous recombination intermediates at R-loops [63]. This complex has been identified as a major regulator of hepatitis B (HBV) and HPV transcription [61,64,65,66,67]. The expression of the EBV protein BNRF1 depletes SMC5/6 by proteasome degradation [68]. Using RNA-seq in BNRF1 knockout EBV lymphoma cells, BNRF1 was identified as an important regulator of lytic DNA replication and late gene expression [68]. SMC5/6 associates with R-loops at the *oriLyt* in the absence of BNRF1 in the lytic EBV cycle. The binding of SMC5/6 to these viral R-loops prevented EBV lytic replication and late viral transcription [68]. The regulation mechanism for the SMC5/6 repression of EBV lytic replication is largely unknown. The SMC5/6 complex has also been identified as an HPV transcription and replication regulator, and future studies are needed to determine if this complex may help regulate HPV R-loop resolution [64,66].

## 4. Kaposi Sarcoma-Associated Herpesvirus (KSHV)

Kaposi’s sarcoma-associated herpesvirus (KSHV) is a double-stranded DNA virus that is associated with Kaposi’s sarcoma, primary effusion lymphoma, and multicentric Castleman’s disease. KSHV is the only human gamma-2 herpesvirus, belonging to the Rhadinovirus genus with a genome size of approximately 165 kb and encoding nearly 100 genes [69]. Most tumor cells contain latent KSHV episomes that have been shown to play a key role in the development of KSHV-associated cancers. Like all herpesviruses, KSHV establishes constant infection in latent and lytic replication. The latent phase is characterized by a persistence in viral episomes for extended periods of time, whereas the reactivation to the lytic cycle results in the expression of the full viral genome and the production of infectious viral progeny [69]. The latency-associated nuclear antigen (LANA) encoded by the ORF73 of KSHV is responsible for episomal maintenance during latency [70].

KSHV induces DNA damage and double-stranded breaks [71,72,73]. The KSHV ORF57 protein is essential for KSHV gene expression and replication [74]. The hTREX (TRanscription-EXport) complex mediates mRNA transcription and nuclear export. Recruitment of hTREX by ORF57 is necessary for export of KSHV mRNAs [75]. Further studies revealed that this interaction leads to double-stranded breaks and DNA damage due to the formation of R-loops [73]. KSHV induced R-loop formation leads to genome instability- a hallmark of cancer.

Using the viral sequences, R-loop-forming sequences (RLFSs) were predicted by overlapping over 6000 viral genomes from double-stranded DNA, single-stranded RNA, single-stranded DNA, and double-stranded RNA viruses [76]. Double-stranded DNA viruses accounted for over 70% of the RLFS-positive genomes. The DNA virus *Ligamenvirales* had no RLFSs in the genome but all viruses in the *herpesvirales* order contained RLFSs [76]. The majority of the RLFSs were located and validated in terminal repeats of the KSHV genomes [76].

## 5. Hepatitis B Virus (HBV)

Hepatitis B virus (HBV) is a small 3.2 kb DNA genome that infects non-dividing liver cells. The viral protein, HBV-encoded oncogene X protein (HBx), is responsible for replication and viral carcinogenesis [77]. HBV activates ATR through an embedded regulatory element (ERE) within the HBx mRNA to complete the replication of its lifecycle [78,79]. Aberrant cellular R-loops trigger ATR activation [80,81]. Although R-loops have not been detected on this genome, the HBV activation of ATR may occur through R-loop formation [79]. As previously described, SMC5/6 associates with R-loops on the EBV genome [68]. HBx degrades SMC5/6 to enhance viral replication and transcription [67,82,83]. It is unknown if SMC5/6 regulates HBV R-loops.

## 6. Conclusions

In this perspective, we highlighted the current understanding of R-loops’ ability to regulate the viral transcription of DNA viruses. R-loops were identified on the viral genomes of HPV, EBV, and KSHV using antibodies that detect RNA–DNA hybrids. For HPV, these R-loops must be resolved to establish persistent infection; failure to do so leads to integration events and the virus is unable to replicate. In contrast, for EBV, the initiation of R-loops on the EBV genome is necessary to initiate viral transcription. Seventy percent of R-loop-forming sequences in over 6000 viral genomes were identified in the genomes of DNA viruses [76]. However, RNA–DNA hybrids have only been validated in a few of the viruses described here. The similarities of HPV, EBV, and KSHV to Merkel cell polyomavirus (MCV) and Adenovirus suggest that the transcription of these viruses is regulated by R-loops. For example, the MCV large T antigen (LT) interacts with the R-loop factor BRD4 during viral transcription and replication [84,85]. BRD4 and the MCV LT complex facilitate MCV replication by replication factor C (RFC1) recruitment to unwind MCV DNA. The importance of these RNA–DNA hybrids in other DNA tumor viruses has yet to be elucidated.

The human genome is composed of 3 billion base pairs and 140 million transcriptional start sites [86]. Three to five percent of the human genome contains R-loops [87], making it difficult to study R-loop licensing mechanisms in mammalian cells. The small 8 kb HPV genome acts like a mini-chromosome, with defined origins of replication and transcriptional start sites. The HPV genome represents a tractable model for examining this fundamental process. The same can be demonstrated for EBV, as R-loop sequences on mini-chromosomes were identified using EBV biology. For example, the EBV replication origin sequence (OriP) replicates once per cell cycle. To determine if R-loops are formed at immunoglobulin class switch regions, these regions were cloned into plasmids containing an OriP and transfected into cells containing EBNA [88]. DNA tumor viruses can be utilized as models to study fundamental R-loop processes.

Although this perspective is focused on DNA viruses, emerging evidence suggests a connection between RNA viruses and R-loops. Unlike DNA viruses, RNA viruses are unable to form R-loops on their genomes. However, virus-induced R-loops can still form during their replication cycles. For example, the human T-cell leukemia virus type 1 (HTLV-1) Tax protein is hyperactivated by NF-κB to increase R-loop accumulation and cause genomic instability (reviewed [89]). Evidence suggests that influenza A and Dengue virus replication can induce cellular apoptosis through R-loop activation [90]. The herpes simplex virus type-1 single-stranded DNA-binding protein (ICP8) can facilitate the development of R-loops in vitro [91]. Human immunodeficiency virus (HIV)-1 may target mammalian R-loops for the integration of its genome [92]. Further evidence has elucidated the importance of using viruses to manipulate host cellular processes through R-loops.

The R-loop field is moving rapidly and consistently discovering new transcriptional mechanisms and processes. How these structures regulate neurological diseases and cancer progression are current areas of study. It is clear that R-loops are important biological regulators and that their importance in human disease has yet to be fully understood. Future studies on DNA viruses and R-loops, either as experimental tools or as novel viral mechanisms, makes this an exciting new area of research.
